# Afoxolaner (NexGard®) in pet snakes for the treatment and control of *Ophionyssus natricis* (Mesostigmata: Macronyssidae)

**DOI:** 10.1186/s13071-022-05611-1

**Published:** 2023-01-06

**Authors:** Jairo Alfonso Mendoza-Roldan, Ettore Napoli, Livia Perles, Matteo Marino, Filippo Spadola, Philippe Berny, Bernadette España, Emanuele Brianti, Frederic Beugnet, Domenico Otranto

**Affiliations:** 1 Department of Veterinary Medicine, University of Bari, Valenzano, Italy; 2 Department of Veterinary Sciences, University of Messina, Messina, Italy; 3 Toxicology Lab, Vetagro Sup., 1 Av Bourgelat, 69280 Marcy L’étoile, France; 4 Boehringer Ingelheim Animal Health, Lyon, France; 5 Faculty of Veterinary Sciences, Bu-Ali Sina University, Hamedan, Iran

**Keywords:** *Ophionyssus natricis*, Snake, Treatment, Afoxolaner, Pharmacokinetic

## Abstract

**Background:**

*Ophionyssus natricis* is the main species of mite that infests captive reptiles. High infestations may result in the host experiencing general discomfort and deleterious effects, even death. Moreover, *O. natricis* is an important vector of reptile vector-borne diseases and is considered to be the putative vector of the *Reptarenavirus,* the causal agent of the inclusion body disease. Despite the cosmopolitan distribution of *O. natricis* in captive reptiles, treatment options are limited. The aim of the present study was to assess the efficacy of afoxolaner (NexGard®; Boehringer Ingelheim, Ingelheim, Germany) in heavily infested, privately owned snakes, evaluate the prevalence of mites and drug availability in the plasma of treated snakes (pharmacokinetics) and perform a clinical examination of animals.

**Methods:**

The study was conducted in two snake breeding facilities, where many snakes were infested with mites. Each animal was clinically examined and weighed, and mite infestations were assessed on the animals and in their enclosures (environment). Animals were treated with a dose of 2.5 mg afoxolaner per kilogram body weight (2.5 mg/kg) administered orally. All animals were examined pre-treatment (T0) and at various time points post-treatment (T1, 6 h; T2, 24 h; T3, 14 days; T4, 28 days). The collected mites were morphologically identified at the species level and the species identity also confirmed molecularly.

**Results:**

Overall, 81 snakes from the two participating facilities (i.e. 70 from site 1 and 11 from site 2) were screened, and 31 (38.3%) snakes were found to have at least one mite. All mites were identified morphologically and molecularly as *O. natricis. Lampropeltis* was the genus of snakes with highest number of infested individuals. Mites were found to be alive on snakes at T1, but at T2 only dead mites were observed, and at T3 and T4 mites were no longer present on the animals or in their environment. No side effects were observed in the treated snakes.

**Conclusions:**

A single oral administration of afoxolaner at 2.5 mg/kg was a safe treatment for snakes and 100% effective for the eradication of natural *O. natricis* infestation without the need to treat the environment of the snake.

**Graphical Abstract:**

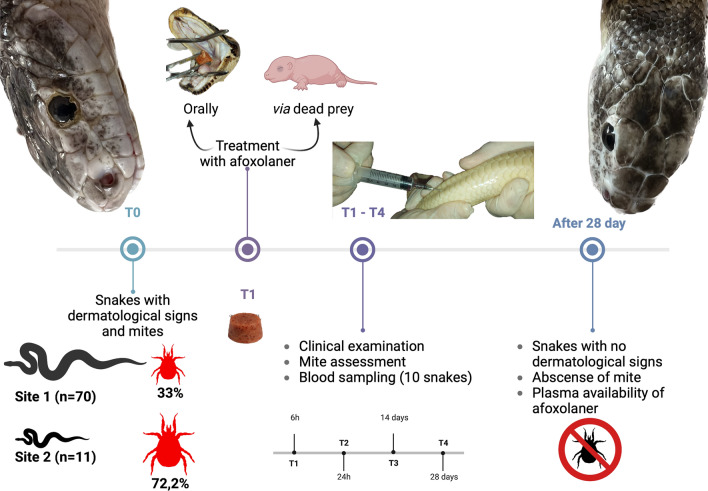

## Background

*Ophionyssus natricis* (Gervais, 1844) (Mesostigmata, Macronyssidae) is the main species of mites infesting captive reptiles of order Squamata (scaled reptiles) [1]. This cosmopolitan species, also known as the snake mite, belongs to the order Mesostigmata, family Macronyssidae, which include many other species of medical and veterinary importance [i.e. *Ornithonyssus bacoti* (Hirst, 1913) from rodents, *Ornithonyssus bursa* (Berlese, 1888) and *Ornithonyssus sylviarum* (Canestrini & Fanzago, 1877) from birds] [2–4]. *Ophionyssus natricis* mainly infests snakes and lizards, yet it prefers the ophidian host (suborder Serpentes: snakes) [5, 6]. Mites have preferred sites of attachment on their reptilian hosts, consisting mainly the anterior part of the body in the gular area, loreal pits and in between the scales in the dorsal area [7]. This species of mite has a life-cycle similar to that of *Dermanyssus gallinae* (De Geer, 1778) (Mesostigmata, Dermanyssidae) from poultry [8, 9], which includes egg, larva, two nymph (i.e. protonymph and deutonymph) and adult stages. Nymphs and adults are hematophagous, feeding on the host and then molting in the environment [1, 10]. The life-cycle can be completed within 7 to 14 days when environmental and host conditions are optimal (i.e. uncleaned terraria, temperatures ranging from 20 °C to 30 °C and > 75% humidity), with high infestation rates within the terrarium or breeding facility [1]. Snake mites have a high motility, allowing them to quickly infest terraria or enclosures [1, 11]. Mites have a painful bite, and high infestations may result in general discomfort to the host, dermatitis, dysecdysis (i.e. improper shedding of the skin or retention), behavioral changes (such as snakes remaining inside the water bowl for long periods of time or constant movement around the enclosure) and even death [12, 13]. High infestation can also have impairing consequences, such as impacts on the loreal pit in vipers [14]. In addition, *O. natricis* is an important vector of reptile vector-borne diseases (RVBDs), such as hemogregarines (i.e. *Hepatozoon* spp.), and it is considered to be the putative vector of a *Reptarenavirus* causing the devastating inclusion body disease (IBD) [15–17]. Importantly, *O. natricis* poses a public health concern due to its vector competence of zoonotic pathogens, being a mechanical vector of *Aeromonas hydrophila*, as well as being found by molecular studies to be positive for *Rickettsia* spp. of the spotted fever group [15, 16]. Additionally, *O. natricis* may develop an unspecific feeding behavior on other host species, such as humans, and thereby have a direct deleterious effect on these hosts, causing dermatitis and increasing the risk of zoonotic transmission of the above-mentioned pathogens [10, 18, 19].

Despite the wide distribution of *O. natricis*, the fastidious and deleterious effects on hosts and the rapid and persistent infestation of reptilian collections, treatment options are limited, and most products currently available for treatment (e.g., dichlorvos, fipronil, pyrethroids) offer a low safety margin [20]. In fact, available treatments are based on a multiple approach targeting adult stages on infested hosts as well as all stages in the environment, with the aim to eliminate sources of reinfestation. In addition, these control approaches should be continuous, taking into account the 3-month survival time of mites in the environment without blood intake [1, 10].

Non-pharmacological methods include temperature increase (> 50 °C) as well as a decrease in humidity (< 50%), both factors which will diminish mite survival, disinfection and cleaning of the enclosure, periodic water baths of the infested animal and isolation and quarantine of infested individuals [1, 12]. Acaricidal treatments include dichlorvos [1], fipronil [21], pyrethroids (e.g. permethrin [22]), selamectin and ivermectin [20]. Nonetheless, these acaricidal treatments have inherent toxicological risks for snakes, as in the case of volatile pyrethrins, pyrethroids, organophosphates and carbamates used in the environment or fipronil applied on the infested animals and not properly washed out [12]. On the other hand, isoxazoline drugs have been successfully used for the oral treatment of *O. natricis* in 20 captive ball pythons (fluralaner) [23] and in two Burmese pythons (afoxolaner) [24]. An oral liquid formulation of fluralaner administered twice at a dose of 0.5 mg/kg body weight at 7 days apart is indicated for the control of *D. gallinae* infestation in poultry farms [25]. *Dermanyssus gallinae* and *O. natricis* belong both to the Dermanyssoidea superfamily of mites and are biologically close. In general, isoxazolines cause an over-stimulation of the nervous systems of invertebrates by blocking the γ-aminobutyric acid (GABA) receptor (GABA-R) and l-glutamate-gated (Glu) chloride channels, which are present at peripheral neuromuscular sites of invertebrates, resulting in hyperexcitation, convulsion and the death of fleas, ticks and mites [26, 27]. Isoxazolines have been shown to have an excellent efficacy against several ectoparasite species with few adverse effects compared to other compounds [28]. NexGard® (Boehringer Ingelheim Animal Health, Boehringer Ingelheim, Ingelheim, Germany) is an oral chewable formulation containing afoxolaner that is labeled to treat and/or prevent infestations with fleas and ticks, as well as demodectic and sarcoptic mange in dogs [29, 30]. In addition, this formulation has been demonstrated to be effective in treating *Otodectes cynotis* ear mites [31].

The present study aimed to assess the efficacy of afoxolaner in two heavily infested, privately owned collections of snakes, evaluate the prevalence of mites and drug availability in plasma (pharmacokinetics) and perform clinical examinations to determine the outcomes after treatment of the animals.

## Methods

### Study sites

**Fig. 1:**
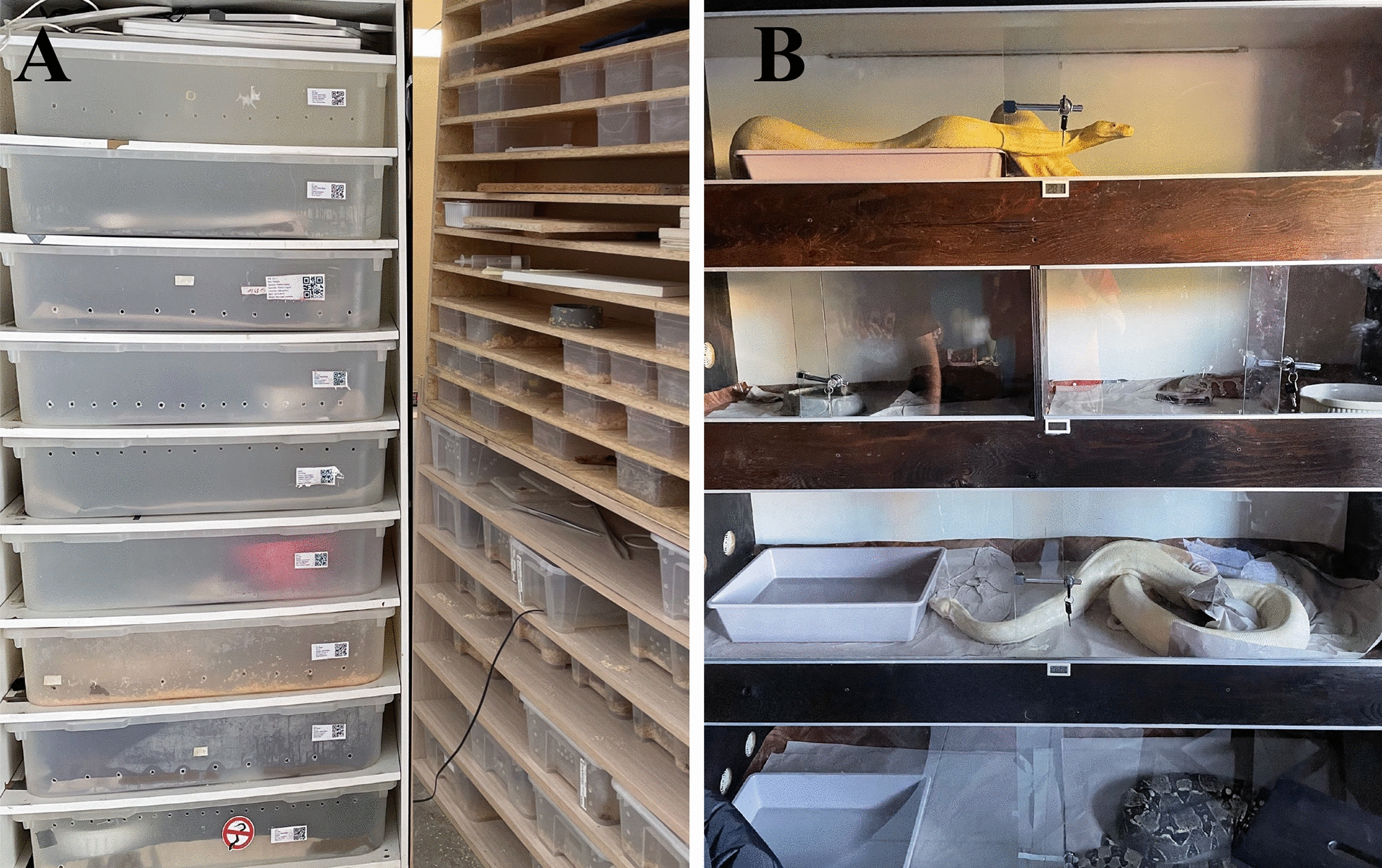
Husbandry conditions of study sites. **a** Site 1, breeding facility with plastic terraria. **b** Site 2, breeding facility with wooden and glass terraria

In May 2022, a male eastern rat snake [*Pantherophis alleghaniensis* (Holbrook, 1836)] (Squamata, Colubridae) infested with *O. natricis* mites was examined at the parasitology unit of Department of Veterinary Sciences, University of Messina (Sicily, Italy). The snake had dermatitis, dysecdysis and ulcerative lesions due to the severe mite infestation. The animal belonged to a breeder that had a snake breeding facility in Caltagirone municipality, Sicily (referred to further as site 1). In this breeding facility snakes were kept separated in plastic terraria in wooden shells, with sawdust as bedding, and fed once a week with frozen mice or chicks (Fig. 1a). The owner reported that many snakes were infested with mites, and he believed that the infestation originated from the introduction of another snake into his facility, a Sumatran short-tailed python [*Python curtus* Schlegel, 1872 (Squamata, Pythonidae)], which he had borrowed from another breeder in the same municipality. This latter site was home to a private collection, with animals kept in wooden and glass terraria and natural decoration and sawdust or soil bedding, where animals were fed also with frozen mice or guinea pigs (referred to further as site 2; Fig. 1b). The snake breeder’s visit to the parasitology unit resulted in both collections being visited at the end of May 2022. The owners were asked to change the bedding for the duration of the study to cardboard or white paper to better visualize the mites.

### Animal screening and afoxolaner administration

**Fig. 2:**
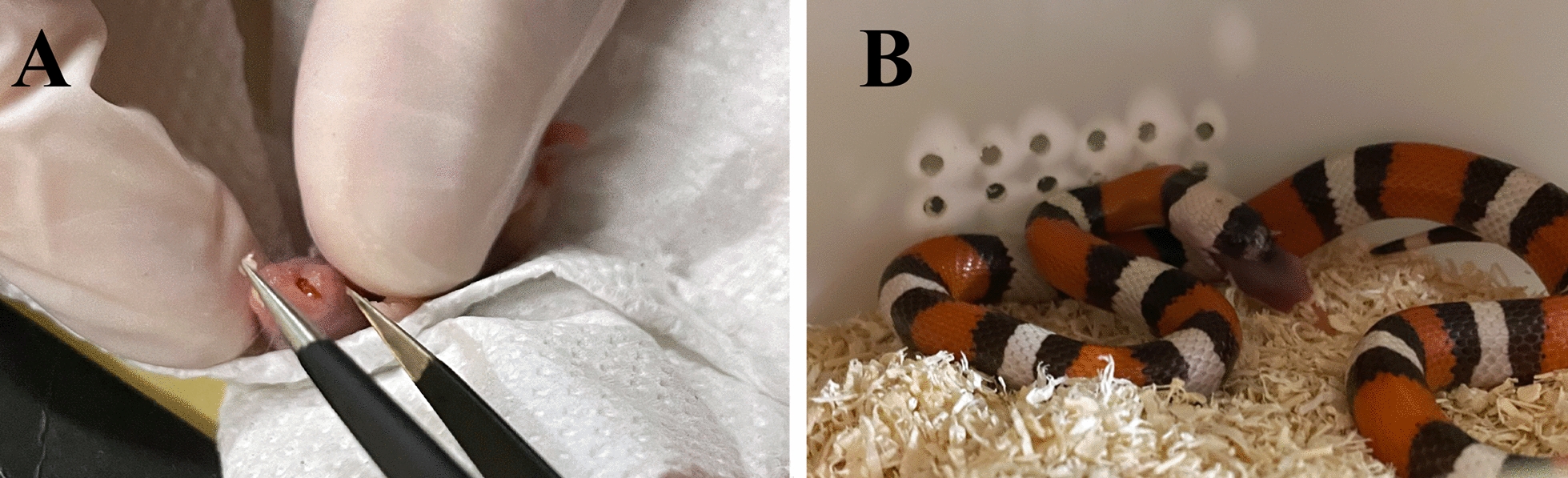
Example of oral administration of afoxolaner to avoid stressful handling in gravid females (**a**) and neonates (**b**) using mice

Each animal was clinically examined and weighed, and all animals and their environments (enclosures) were assessed for mite infestations. All animals were treated with afoxolaner (NexGard®), administered orally, at a dose as close as possible to 2.5 mg/kg based on the animal’s weight, using 68 mg [intended for medium to large dogs (10–25 kg)] or 11.3 mg [intended for small dogs (2–4 kg)] per tablet. To avoid stressful handling, gravid females and neonates were fed with dead mice containing the afoxolaner dose in their mouths(Fig. 2). To assess the parasitic load of mites, descriptive statistics was calculated using Quantitative Parasitology software, version 3.0 [32]. Prevalence, mean abundance (i.e. number of mites per total number of hosts) and mean intensity (i.e. number of mites per number of infested hosts) of infestation were calculated. Animals were examined pre-treatment (T0) and at various post-treatment time points (T1, 6 h; T2, 24 h; T3, 14 days; T4, 28 days). No other environmental treatments were done to assess afoxolaner efficacy and avoid any interaction.

### Ectoparasite identification

Mites were collected and stored in microtubes in absolute ethanol. Some of the mites were clarified and slide-mounted in Hoyer’s medium [33]. Dichotomous keys [34, 35] as well as original species descriptions [36–38] were used for morphological identification of Mesostigmata mites of the family Macronyssidae.

Prior to the mites being mounted on slides, DNA was extracted based on lysis with the guanidine isothiocyanate protocol (GT), adapted from Chomczynski [39]. This protocol was adapted to avoid mite destruction, which allowed the preservation of a voucher [40]. Extractions were performed from individual mites.

PCRs of mites were performed to confirm species identity by a molecular method. Primers of the gene for 18S rRNA (18S+ and 18S−, respectively), which amplify a fragment of 480 bp of the V4 region [41], were used for the PCR assays. The cycling conditions for the PCRs were: an initial denaturation at 94 °C for 1 min, then 30 cycles of 20 s at 94 °C, 50 °C for 30 s and 72 °C for 1 min and 30 s, with a final cycle at 25 °C. Amplified DNA was subjected to electrophoresis in a 2% agarose gel stained with GelRed (VWR International PBI, Milan, Italy) and viewed on a GelLogic 100 gel documentation system (Kodak, Rochester, NY, USA). Amplicons were purified using 10 μl of PCR product mixed with 0.5 μl of *Escherichia coli* exonuclease I (*Exo*I; Fermentas Life Sciences, Thermo Fisher Scientific, Waltham, MA, USA), 1 μl of shrimp alkaline phosphatase (SAP) and 0.5 μl of SAP reaction buffer (Fermentas Life Sciences, Thermo Fisher Scientific) to remove unused primers and unincorporated dNTPs. This mix was incubated at 37 °C for 20 min; following enzyme inactivation, it was incubated at 85 °C for 15 min. PCR-purified products were sequenced using the Taq Dye Doxy Terminator Cycle Sequencing Kit (v.2; Applied Biosystems, Thermo Fisher Scientific) in an automated sequencer (model ABI-PRISM 377; Applied Biosystems, Thermo Fisher Scientific). Sequences were analyzed by Geneious version 11.1.4 software and compared with those available in Genbank database by the Basic Local Alignment Search Tool (BLAST) [42].

### Pharmacokinetics of afoxolaner in snakes

A specific analytical technique was developed with liquid chromatography and tandem mass spectrometry (LC-MSMS). This technique was based on that developed by Kilp et al. [43].

Plasma samples (0.2 ml) were extracted using 100 mg QuEChERS salts (4 g MgSO_4_ and 1 g NaCl) and 0.275 ml acetonitrile. Each sample was spiked with an internal standard (fluralaner) at a constant concentration for quantification purposes. All samples were mixed for 1 min and centrifuged at 8000 *g* for 5 min. The supernatant layer was collected and used for analysis.

The LC-MSMS apparatus was the Agilent 1260 Affinity II Prime LC system equipped with a 6470A triple quadrupole mass spectrometer (Agilent Technologies France, Les Ulis, France). The column used was a VWR Lichrospher 100 C18e (100 mm × 4 mm ID, dp 5 µm). The mobile phase consisted of a mixture of acetonitrile and water (with solution A: 10 mM ammonium carbonate, pH 9) and was delivered at 0.5 ml/min using a gradient elution schedule (from time 0 to 5 min: water 70%, solution A 30%; 5 min: water 90%, solution A 10%; 5.5–11 min: water 70%, solution A 30%). The total run time was 11 min. Afoxolaner was detected using the negative ion mode (precursor ion, 3 product ions). The method was validated according to current standards. In order to cover for matrix effects, samples were quantified by comparison with spiked samples on the same day. Linearity was checked between 2 and 100 µg/l and accepted only if *r*^2^ > 0.99.

## Results

**Fig. 3:**
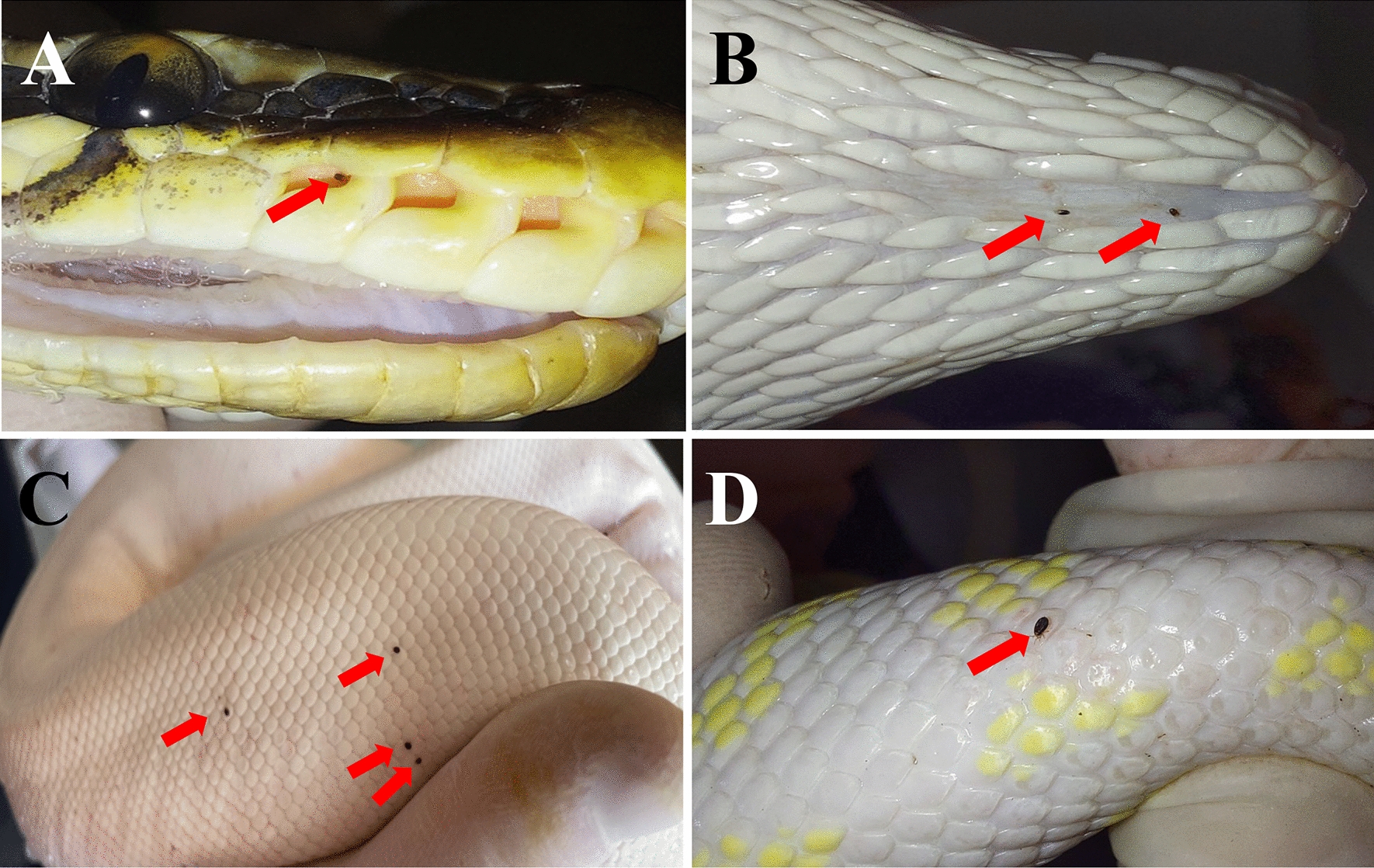
*Ophionyssus natricis* (snake mite) infestation in observed snakes. **a** A mite in the loreal pit, **b** mites in the gular area, **c** mites on dorsal scales of a *Python regius*, **d** a mite on the dorsal region of a *Lampropeltis getula*

**Fig. 4:**
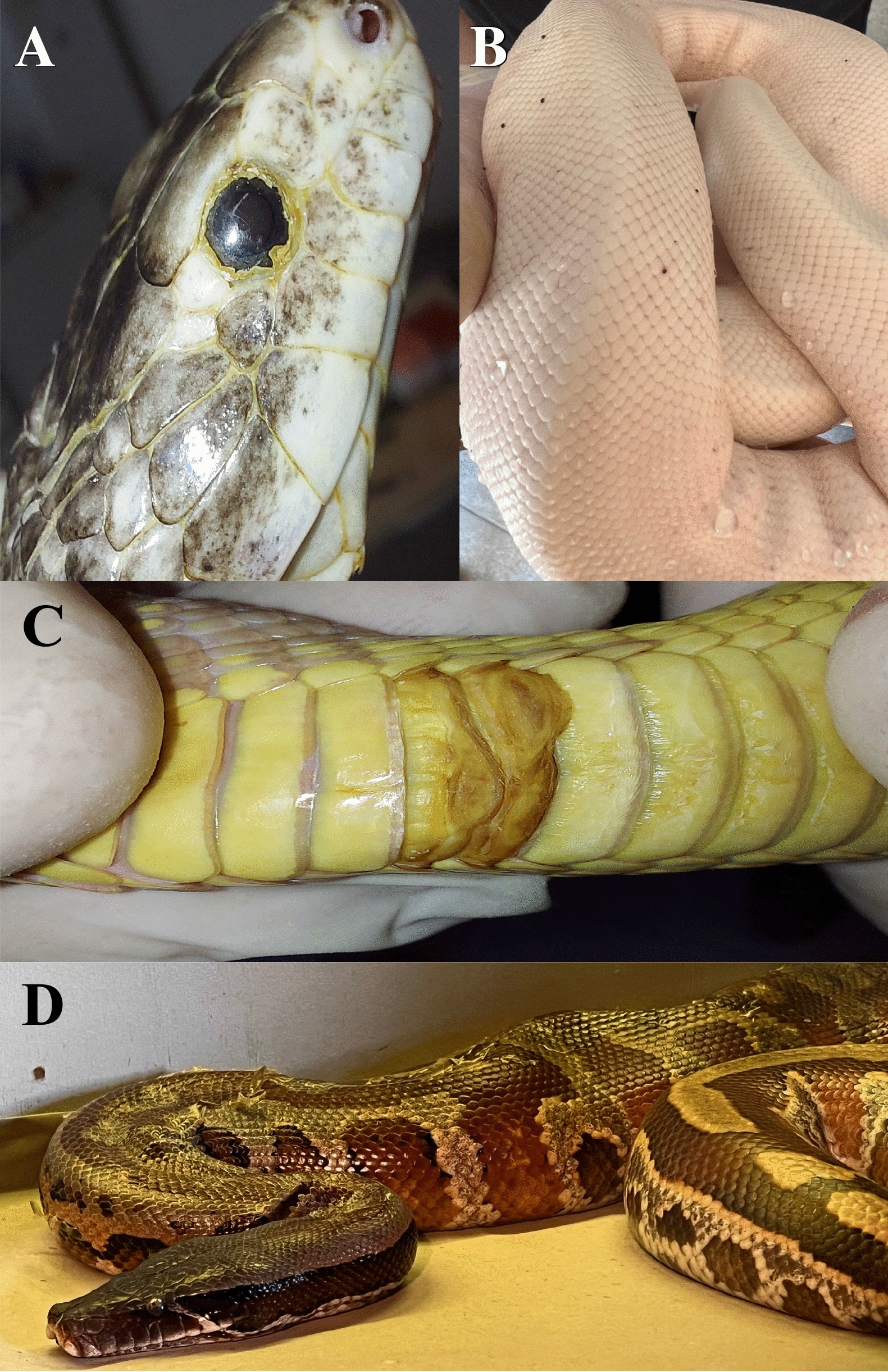
Dermal lesions associated with mite infestations in snakes. **a** Dermatitis and dysecdysis in *Pantherophis alleganiensis*, **b** erythema in leucistic *Python regius*, **c** ulcerative lesion in ventral scale of *Lampropeltis getula*, **d** dysecdysis in *Python curtus*

A total of 81 snakes from the two breeding facilities were screened, 70 from site 1 and 11 from site 2. These snakes belonged to the Boidae, Colubridae and Pythonidae families, and were represented by six genera (i.e. *Boa, Epicrates, Lampropeltis*, *Pantherophis*, *Python*, *Antaresia*) and 16 species (Table 1). The body weight of each snake varied from 0.025 kg (14 neonates) to 11.5 kg (one *Boa constrictor imperator*), with an overall mean [± standard deviation (SD)] body weight of 1 ± 3 kg. In terms of sex, 42 were female (3 of which were gravid) and 39 were male. Of the 81 animals, 31 were found to have at least one mite [38.3%; 95% confidence interval (CI): 27.6–49.7%; Fig. 3]. The mean infestation intensity was 8.4 (95% CI: 6–13.2) and mean abundance was 3.22 (95% CI: 2–5.4). Snakes of genus *Lampropeltis* accounted for highest number of infested individuals (*n* = 14). Ten snakes presented with at least one clinical sign related to the mite infestation (i.e. erythema, dysecdysis, dermatitis; Fig. 4). The infestation prevalence at site 1 and site 2 was 33% (23/70) and 72.7% (8/11), respectively.

**Table 1:** Species and number of snakes sampled per site, with infestation rates and number of animals from which blood samples were collected

Site^a^	Species of snake examined	Number of snakes examined	Sex of snake examined	Weight of snake examined (kg)	Tablet size of afoxolaner used	Number of infested snakes; number of mites	Observation	Number of animals from which blood samples were collected
Site 1	*Lampropeltis getula californiae*	14	7F; 7M	0.025–0.8	11.3	5; 5–10	One gravid; One neonate	3
	*Lampropeltis getula floridana*	7	3F; 4M	0.025–0.65	11.3	3; 1–5	One neonate	1
	*Lampropeltis getula nigrita*	2	2F; 0M	0.23–0.5	11.3	1; 1		
	*Lampropeltis getula meansi*	2	1F; 1M	0.025–0.127	11.3	0	One neonate	
	*Lampropeltis triangulum campbelli*	22	11F; 11M	0.025–0.4	11.3	3; 5	Two gravid; four neonates	1
	*Lampropeltis triangulum sinaloe*	5	3F; 2M	0.024–0.4	11.3	1; 1	Two neonate	1
	*Lampropeltis triangulum hondurensis*	1	1F; M0	0.025	11.3	0	One neonate	
	Mexicorn R1	3	2F; 1M	0.025–0.2	11.3	0	One neonate	
	*Pantherophis alleghaniensis*	1	M	1.16	11.3	1; 20	Dermatitis, dysecdysis	1
	*Pantherophis guttatus*	6	3F;3M	0.025–0.3	11.3	1; 5	One neonate; one scaleless	
	*Python brongersmai*	2	2F	6.4–13	68	1; 10	Dermatitis	2
	*Python molurus*	1	1F	10	68	1; 10	Dermatitis, erythema	1
	*Python regius*	4	3F; 1M	0.9–2	11.3	4; 3–50	Dermatitis, erythema; dysecdysis	
Site 2	*Antaresia childreni*	2	1F; 1M	0.025	11.3	0	Neonates	
	*Boa constrictor constrictor*	1	1M	4	68	1; 5	Dermatitis	
	*Boa constrictor imperator*	4	2F; 2M	0.1–12	11.3–68	3; 5–50	Dermatitis, erythema; dysecdysis	
	*Epicrates cencrhia*	1	1M	0.86	11.3	1; 10		
	*Lampropeltis triangulum sinaloae*	1	1M	0.92	11.3	0		
	*Python brongersmai*	1	1M	7	68	1; 10		
	*Python regius*	1	1M	1.8	11.3	0		

*F* Female,* M* male

^a^Site 1, breeding facility with plastic terraria. Site 2, breeding facility with wooden and glass terraria

Following treatment, mites were alive at T1, while at T2 only dead mites were observed. From T3 up to T4 mites were no longer observed on animals nor in the environment. No dermatological signs were recorded at T4. The only gravid female (*Lampropeltis getula californiae*) and neonate (*Lampropeltis triangulum sinaloae*) infested were negative for mites after T2. No related adverse events were recorded after oral administration of afoxolaner in any of the snakes.

**Fig. 5:**
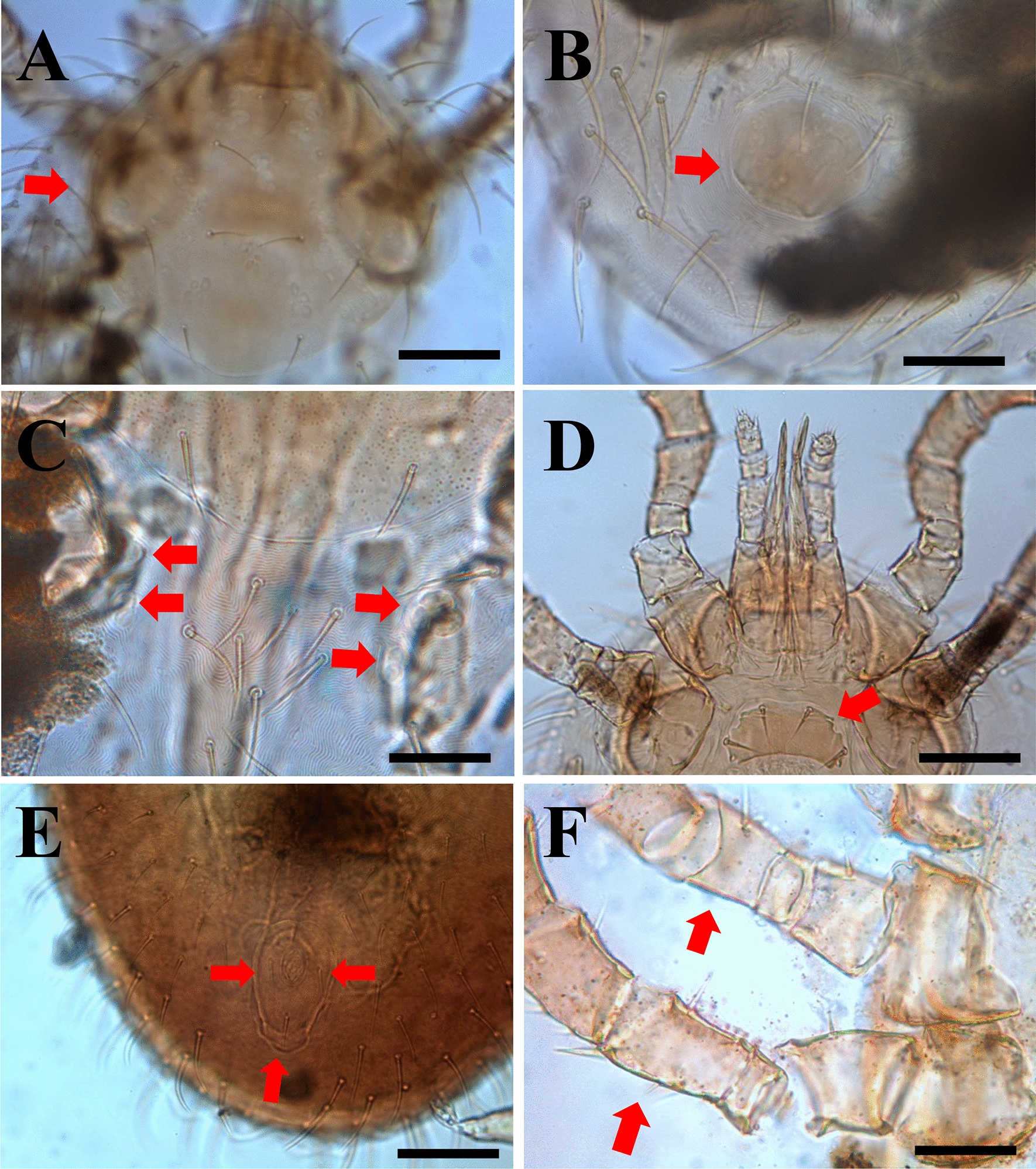
Key morphological features of the snake mite *Ophionyssus natricis*. **a** Female podonotal shield (red arrow) with 10 pairs of setae, **b** Female with minute pygidial shield (red arrow) without setae, **c** female with two pairs of minute mesonotal scutellae (red arrows), **d** Female sternal shield (red arrowe), **e** female anal shield with three setae (red arrows), **f** male femur III and IV without modified ventral setae, and femur III spur absent (red arrows); Scale bars: 100 μm (**a**, **b**, **d**, **e**); 50 μm (**c**, **d**, **f**)

Mites were identified as *O. natricis* through morphological diagnostic features. These included female features, such as a large dorsal anterior shield (podonotal) with 10 pairs of setae (Fig. 5a), minute pygidial shield without setae (Fig. 5b), with two pairs of minute mesonotal scutellae (Fig. 5c), a sternal shield (Fig. 5d) with a width/length ratio of 2.5 and an anal shield with three setae (Fig. 5e). In males, the holoventral shield was absent, and the sternogenital region had two pairs of setae. Male femur III and IV were without modified ventral setae, and the femur III ventral spur was absent (Fig. 5f). Mite species was also confirmed by a molecular method through BLASTn analysis, with an identity of 100% (accession number: OP752168) with sequence MT163329 of *O. natricis* from Mexico, and 99.8% (accession number: OP752167) with sequence FJ911853 of *O. natricis* from the USA.

### Pharmacokinetics of afoxolaner in ten snakes

**Fig. 6:**
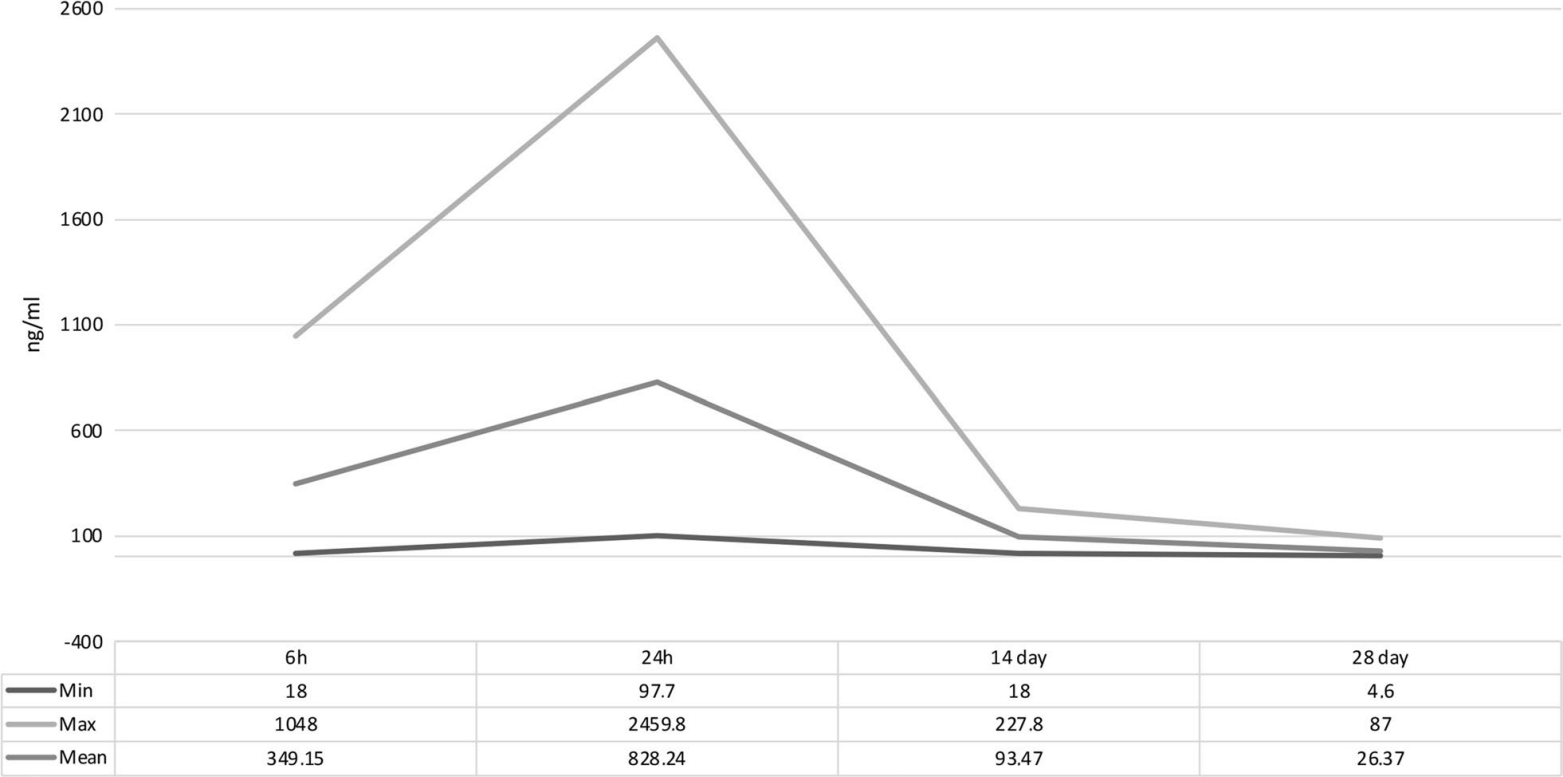
Plasma concentration levels of afoxolaner from 10 sampled snakes. Minimum (Min), maximum (Max) and mean values (ng/ml) are given for the four time points

Afoxolaner availability in the plasma was found at different concentrations in samples collected from the 10 snakes sampled after 6 h up to day 28 (Table 2; Fig. 6). At 6 h post oral administration (T1), all sampled snakes had plasma afoxolaner concentrations ranging from 18 to 1048 ng/ml, with a mean value of 349.15 ng/ml. Plasma concentrations of afoxolaner increased in seven of the 10 snakes from 6 to 24 h post oral administration. Of the six *Lampropeltis* spp. snakes treated, three had lower plasma concentrations of afoxolaner at T2 (24-h time point; Table 2). After the 24-h peak, afoxolaner concentrations decreased in all snakes. All snakes had detectable plasma concentration levels of afoxolaner at day 28 (i.e. T4; mean of 26.37 ng/ml) ranging from 4.6 ng/ml in a *Lampropeltis getula californiae* to 53.4 ng/ml in a *Python curtus* (Fig. 6).

**Table 2:** Afoxolaner concentrations in plasma of 10 sampled snakes in four time points

Animal data				Post administration time point (ng/ml)			
Snake ID	Species	Sex	Weight (kg)	6 h	24 h	14 day	28 day
S01	*Pantherophis alleganiensis*	Male	1.163	289.4	877.1	101	11.6
S06	*Python molurus*	Female	10	18	446.9	94.1	22.7
S07	*Python curtus*	Female	6.4	113.2	664.3	227.8	53.4
S08	*Python curtus*	Female	13	38	973.7	129.9	22.6
S15	*Lampropeltis triangulum campbelli*	Male	0.323	1048	853.5	135.8	9.8
S17	*Lampropeltis triangulum sinaloe*	Male	0.331	983.3	2459.8	96.2	87
S19	*Lampropeltis getula californiae*	Male	0.283	151.8	139.8	18	6.7
S23	*Lampropeltis getula californiae*	Male	0.279	289.4	97.7	25.4	4.6
S26	*Lampropeltis getula californiae*	Male	0.326	439.2	747	60.3	9.5
S28	*Lampropeltis getula floridana*	Male	0.492	121.2	1022.6	46.2	35.8
		Arithmetic mean		349.2	828.2	93.5	26.4
		Standard deviation		354.8	625.3	59.1	24.8

## Discussion

The results of the present study show that a single oral administration of afoxolaner at a dose of 2.5 mg/kg is safe and effective in snakes for the treatment of a natural *O. natricis* infestation without the need of environmental measures. The acaricidal efficacy of this dose of afoxolaner is also supported by the detection of this drug in the plasma of the treated snakes up to 28 days post oral administration, which is close to that described in dogs. Overall, the results suggest that regular administration (monthly) of afoxolaner to captive snakes can prevent future infestations with this mite. In addition, this study confirmed the high prevalence of *O. natricis* in breeding facilities where mites infested all types of snakes kept in these facilities, irrespective of species, size or type of scales.

*Ophionyssus natricis* has been previously reported in reptile collections in Italy, infesting constrictor snakes (Boidae—*Boa constrictor*, *Eunectes murinus*; Pythonidae—*Morelia spilota*) [40], as well as Boid (*Epicrates cencrhia*), Pythonid (*Python bivittatus*, *Python regius*) and Colubrids snakes (*Pantherophis guttatus*). A previous survey found the prevalence of this mite to reach as high as 65.8% (i.e. 25/38) in central and northern Italy [44]. The mite prevalence in site 2 (i.e. 72.7%) was similar to that described in this previous survey [44]. Indeed, the prevalence of these mites as well as the infestation rates vary depending on the size of the breeding facility, taking into account that these mites can infest rapidly and efficiently all terraria. The prevalence has been shown to be lower (i.e. 3.4%; 1/29) in cases of co-infestation with other Mesostigmata mites [13]. The overall prevalence of the infestation reported in the present study was similar to that recorded in a previous survey in snake collections from Belgium (i.e. 40%) [45], which was classified as a heavy infestation. The indiscriminate feeding behavior of *O. natricis* on any type of snake was observed in the present study. In other studies, this mite species has often been associated with constrictor families of snakes [5, 6, 46], with only a few case reports of infestation on colubrid snakes [47, 48] and some lizards, such as *Pogona vitticeps* [7]. Although *O. natricis* has been recorded feeding on humans [18], despite the close proximity of the owners with their snakes in the two study sites in the present study, none of the owners and relatives complained about bites. Given that all animals were fed with frozen prey, the most likely source of the mite infestation may have been the introduction of a snake to site 1 that had been recently acquired by the owner of site 2. Bedding from the environment can also be a source of mites, as mites can survive for long period of time without feeding [1], which makes it important to sanitize or freeze bedding prior to use in order to avoid future infestations. Accordingly, prevention and control of snake mites is of pivotal importance to avoid the direct deleterious effects on the infested snake and the possibility of transmission of diseases.

The single dose of oral afoxolaner, used for treatment and prevention, was efficacious when delivered directly into the mouth, as previously recorded for *Python molurus bivittatus* [24], or when given with a prey as food. This latter modality of drug delivery could facilitate the treatment of animals. Exactly as has been described for fleas and ticks, the rapid absorption of afoxolaner, which was detected in the plasma at 6 h post oral administration, provided a curative efficacy by killing mites already attached and feeding on their host. The persistency of afoxolaner for several weeks after administration provided a sustained efficacy, as shown in the plasma concentration, enabling the killing of mites that were in the environment at the time of drug administration but subsequently attempted to infest the treated snakes. Therefore, the rapid onset and availability in the plasma up to T4 (28 days) stopped the life-cycle of the mites and provided decontamination of the environment [1]. Afoxolaner pharmacokinetic studies in dogs administered a dose of 2.5 mg/kg demonstrated a rapid drug absorption (C_max_: 2–4 h), high bioavailability (> 70%), moderate distribution into tissues and low systemic clearance (i.e. terminal plasma half-life of approximately 2 weeks), therefore ensuring that the drug plasma level was sufficent for efficacy against ectoparasites over a 1-month period [49]. This study also demonstrated that the 90% lethal dose (LD_90_) against fleas was around 20 ng/ml when counts were made at 24 h, and 110 ng/ml against *Dermacentor* and *Rhipicephalus* tick species when counts were made at 48 h [49]. It was not possible to calculate the LD_90_ in the present study, but the LD_90_ is related to the time of feeding (correlated to the quantity of active ingredient ingested), and a very low concentration may be sufficient to kill arthropods feeding for several days. Although only blood samples were collected from 10 snakes for testing the plasma concentration of afoxolaner, the results suggest that the plasma concentration was still sufficiently high at 28 days post oral administration to avoid new mite infestation in the treated snakes.

## Conclusion

The data of the present study confirm the efficacy of afoxolaner administered as a single oral dose to eradicate *O. natricis* in infested collections of snakes. In addition, this treatment can be used to treat recently acquired snakes that must go through quarantine as an initial ectoparasitic treatment. The pharmacokinetic properties of afoxolaner in the treated snakes were close to what is described in dogs. The results from this study suggest that a monthly administration of afoxolaner given directly or *via* prey to captive snakes can control future infestations with this zoonotic mite.

## Data Availability

Sequences were deposited in GenBank (accession numbers: OP752167, OP752168).
